# A Comparison of Thoracolumbar Injury Classification in Spine Trauma Patients Among Neurosurgeons in East Africa Versus North America

**DOI:** 10.7759/cureus.31761

**Published:** 2022-11-21

**Authors:** Caitlyn J Smith, Eyerusalem B Bergene, Abraham Tadele, Fassil B Mesfin

**Affiliations:** 1 Neurological Surgery, University of Missouri School of Medicine, Columbia, USA; 2 Neurosurgery, Millennium School of Medicine, Addis Ababa, ETH; 3 Orthopedics, Neurosurgery - Spine, University of Missouri School of Medicine, Columbia, USA

**Keywords:** aans, regional practice, survey, trauma, thoracolumbar injury, north america, africa, denis 3-column, ao, tlics

## Abstract

Background

In January 2021, we published findings evaluating the validity of thoracolumbar injury classification and biomechanical approach in the clinical outcome of operative and non-operative treatments. A notable result in our study was patients with unstable burst fractures received an Arbeitsgemeinschaft für Osteosynthesefragen System (AO) score that recommended conservative treatment compared to a Thoracolumbar Injury Classification and Severity Scale (TLICS) score that recommended surgical intervention. We designed a survey to determine reported differences in thoracolumbar injury classification, including the percentage of thoracolumbar spine fractures, type of classification system(s) used, use of classification system by board-certified neurosurgeons and neurosurgical residents, reliance on classification system to guide management, use of MRI in the evaluation of the posterior ligamentous complex, and readmission rate < 90 days at treating facilities. This study aims to determine which areas of neurosurgical practice in spine trauma patients differ among surgeons in North America and East Africa, including Ethiopia, Kenya, and Sudan. Multiple classification systems have been proposed to describe thoracolumbar spine injuries. We hypothesized that there would be marked variability in the classification systems used to evaluate thoracolumbar spine injury among neurosurgeons in North America and East Africa.

Methods

The survey consisted of seven questions and was sent to 440 neurosurgeons practicing on the continents of North America and East Africa.

Results

A total of 67 surgeons responded, 50 from North America and 17 from East Africa, including Ethiopia, Kenya, and Sudan. A significant percentage of African respondents reported a higher thoracolumbar spine fracture rate than respondents in North America (53% and 30%, respectively). Regarding the classification system used, 65% of surgeons in East Africa reported using TLICS, whereas 62% of surgeons in North America reported using Denis 3-column classification. For patients with spine trauma, surgeons in East Africa and North America reported a similar percentage of readmission <90 days (47% and 52%, respectively).

Conclusion

Our findings vary in spine trauma classification for American and East African patients and still highlight crucial areas for improvement due to patient load, education, and resource accessibility.

## Introduction

Traumatic spine injury is a severe condition associated with everlasting disability or even mortality. Spinal injuries lead to significant physical, emotional, and financial burdens on affected individuals, families, and society. The outcome after a traumatic spinal injury is far better in developed countries than in low-income countries because of the availability of high-quality pre-hospital management, treatment, rehabilitation, and long-term support facilities for disabled patients [1]. Such resources are generally limited in developing countries; thus, establishing medical priorities can be difficult. Information about epidemiology and outcome after spine injuries is necessary to develop appropriate prevention and treatment strategies in each country. Such information is scarce, however, in low-income countries [2-3].

We previously evaluated the validity of thoracolumbar (TL) injury classification and biomechanical approach in the clinical outcome of operative and non-operative treatments. Despite numerous methodologies for assessing patients with TL injuries with a TLICS score of 4 or a "grey zone" score, the standardized classification and treatment of TL spine injuries remain controversial. It was hypothesized that there would be variability in how patients are evaluated among treating physicians with current TL scoring systems, Arbeitsgemeinschaft für Osteosynthesefragen System (AO), and Thoracolumbar Injury Classification and Severity Score (TLICS). Our study found that patients with unstable burst fractures received an AO score that recommended conservative treatment. For example, patients without neurological deficits (n=19/37) received an AO score between 1 to 3 points in which AO recommends conservative treatment, compared to patients (n=37) who received a TLICS > 4. In the guidance of neurosurgical management, TLICS may be more reliable compared to AO recommendations in managing unstable burst fractures without neurological deficits, as the AO scoring system recommended conservative treatment in this study. However, this conclusion needs to be further evaluated through multicenter prospective studies. The results of this study surprised us in that we expected to find a less extreme variability in how patients with spine trauma were scored using thoracolumbar spine injury classification systems.

Based on these findings, we created a questionnaire sent to treating neurosurgeons in North America and East Africa to further evaluate the evaluation of thoracolumbar injury and patient outcomes treated surgically and non-surgically. Our current findings in this recent study encouraged us to advance neurosurgical practice in East Africa. As of 2010, two board-certified neurosurgeons who completed training outside of Ethiopia were practicing in Addis Ababa, Ethiopia. The capital city, Addis Ababa, has a population of over 6 million individuals [4]. In 2010, 100-150 neurosurgical procedures were performed annually in Ethiopia [4]. However, it is evident from some responses that there is still room for improvement in eliminating the shortcoming of the proposed classification systems used in the evaluation of thoracolumbar spine injuries, increasing the numbers of board-certified neurosurgeons in East Africa, improving the infrastructure of treating facilities. Lack of resources, including operative space, post-operative recovery hospital space, instrumentation for the surgical procedure, neurosurgery training programs, and trained medical staff, are critical factors likely driving differences in outcomes of spine trauma patients.

Given the previously reported differences in outcomes and resource accessibility among neurosurgery departments in East Africa and North America, we hypothesize that there will be significant reported differences in the evaluation of thoracolumbar spine injury due to the use of different classification systems by treating neurosurgeons, specifically, in terms of the percentage of thoracolumbar spine fractures, type of classification system(s) used, use of classification system by medical trainees, reliance on classification system to guide management, use of MRI in the evaluation of the posterior ligamentous complex (PLC), and readmission rate <90 days at treating facilities. We hypothesize that the difference will involve different factors, including resource accessibility, neurosurgical training, education, hospital infrastructure, and patient load.

## Materials and methods

Our survey was administered via the Qualtrics test (University of Missouri, USA). A total of seven questions were included. The questions, answer choices, and responses are reported in the results section. The study was reviewed and approved by the Institutional Review Board University of Missouri - Columbia (approval number: 378524).

The most common injuries to the spinal column are Thoracolumbar (TL) fractures. In the United States, the annual incidence of TL spinal injuries is approximately 15,000, whereas high-speed motor vehicle accidents in younger patients account for the majority of the cases [5]. Different thoracolumbar spine injury classification systems have been proposed. However, no single classification system has been established as the superior system standardizing treatment approaches or facilitating proper communication among treating physicians. Previous studies have reported moderate repeatability of the Denis and AO systems [6]. In the evaluation of the proposed AO classification system, there was a reported inter-observer agreement of 67% for the three injuries (types A, B, C), and the inclusion of 53 different injuries led to a marked reduction in reliability [7]. Among the proposed classification systems, the authors selected thoracolumbar injury classification as the question to evaluate the department's neurosurgical training and resource accessibility from which the respondent operates, given that it is well established and is used widely in most countries, compared to the other classification systems.

The survey was released on October 24, 2021, and selected neurosurgical departments and centers in North America, including the West and East Coasts of the United States and the Midwest and Canadian surgeons. We utilized the American Academy of Neurological Surgeons (AANS) directory to obtain the email addresses of neurosurgical faculty. The listed second author provided a correspondence list of the respondents practicing in East Africa, including the countries of Ethiopia, Kenya, and Sudan. Those email addresses for surgeons practicing in East Africa were obtained via an extensive correspondence list by the second author. The survey was emailed weekly over three months (October 24, 2021 to December 24, 2021). When the survey was opened or completed, the primary author of this study was notified.

## Results

**Figure 1:**
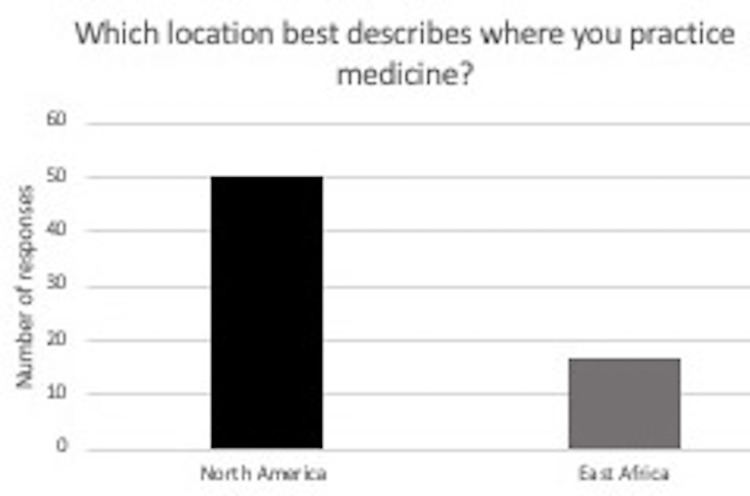
In response to the question “Which location best describes where you practice medicine?” and given the option of North America or East Africa, 50 responded as North America, 17 as East Africa.

**Figure 2:**
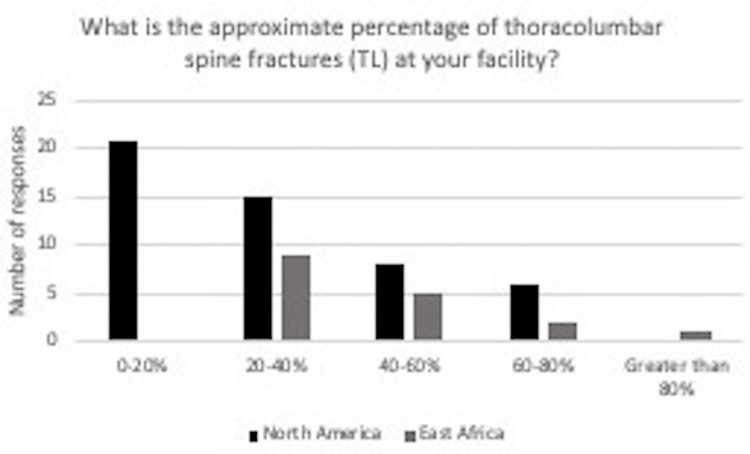
In response to the question “What is the approximate percentage of thoracolumbar spine fractures at your facility?” for surgeons in North America, 21 said 0-20%, 15 said 20-40%, eight said 40-60%, six said 60-80% and 0 said greater than 80%. For surgeons practicing in East Africa, 0 said 0-20%, nine said 20-40%, five said 40-60%, two said 60-80%, and one said greater than 80%.

**Figure 3:**
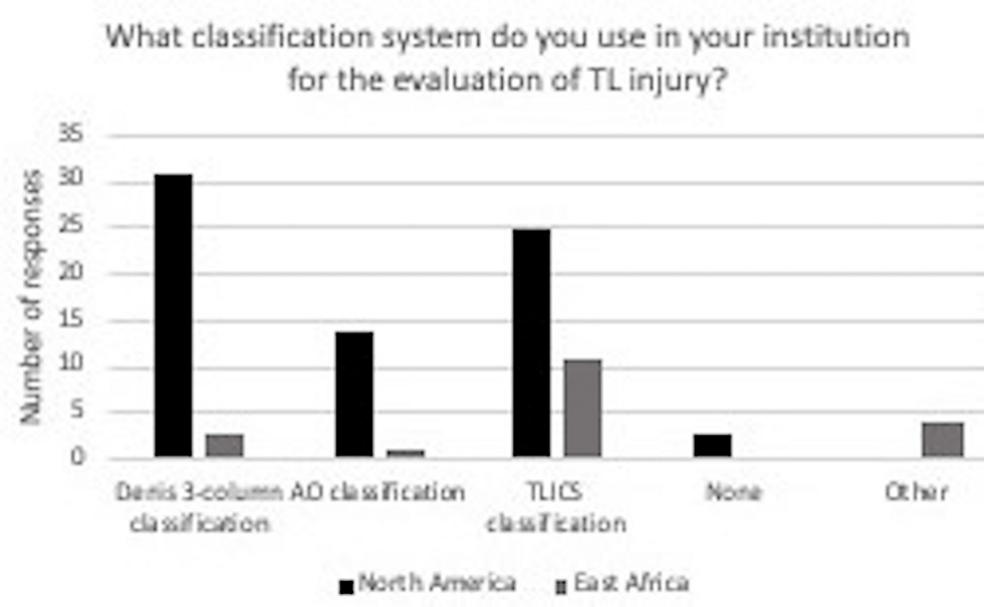
In response to the question “What classification system(s) do you use in your institution for the evaluation of thoracolumbar injury?” for surgeons in North America, 31 said Denis 3-column classification, 14 said AO classification, 25 said TLICS classification, three said none and 0 said other. For surgeons in East Africa, three said Denis 3-column, one said AO classification, 11 said TLICS classification, 0 said none, and four said other, and all specified the Magerl classification system. AO: Osteosynthesefragen System; TLICS: Thoracolumbar Injury Classification and Severity Scale.

The survey was emailed to a total of 440 practicing neurosurgeons. Twelve, or 2.7%, opted out of the study. One hundred and twenty-five were never opened. Of the 239 that were opened, 66 were completed. Of those who completed, as shown in Figure 1, 50 said they practiced in North America, and 17 said they practiced in East Africa (11 Ethiopia, 3 Kenya, and 2 Sudan). Most spine trauma cases at the respondent’s treating facility ranged from 0 to 20% in North America and 20 to 40% in East Africa (Figure 2). The Denis 3-column and TLICS classification systems were the most common classification systems used by treating surgeons in North America (Figure 3). The TLICS classification system and other classification categories were the most common classification systems used by spine trauma surgeons from East Africa (Figure 3). Of the four classification systems reported in the other category by the East African surgeons, all were specified as the Magerl classification system (Figure 3).

**Figure 4:**
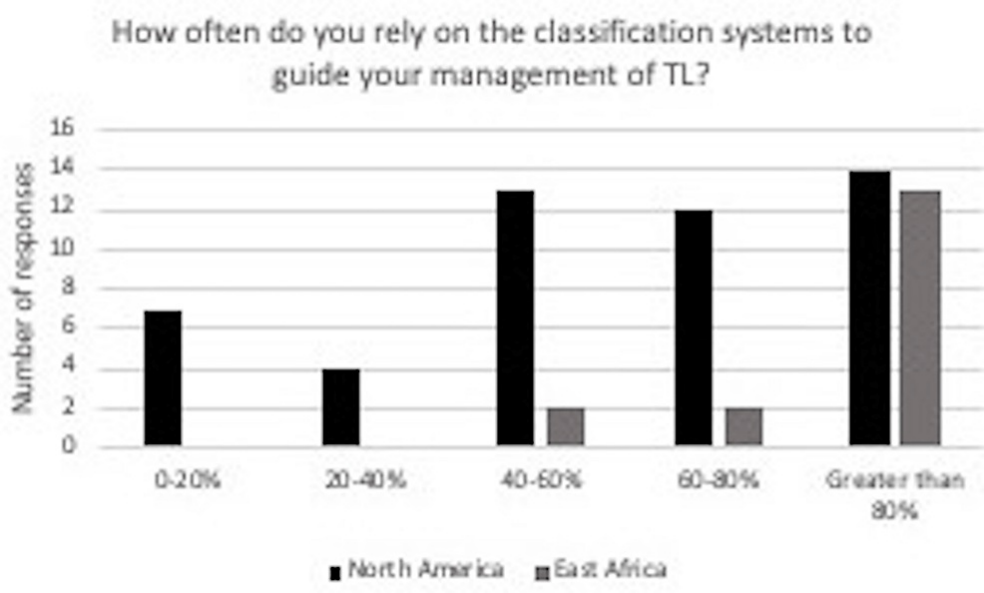
In response to the question “How often do you rely on the classification systems to guide your management of thoracolumbar injury?” for surgeons in North America, seven said 0-20%, four said 20-40%, 13 said 40-60%, 12 said 60-80%, 14 said greater than 80%. For those practicing in East Africa, 0 said 0-20%, 0 said 20-40%, two said 40-60%, two said 60-80%, 13 said greater than 80%.

**Figure 5:**
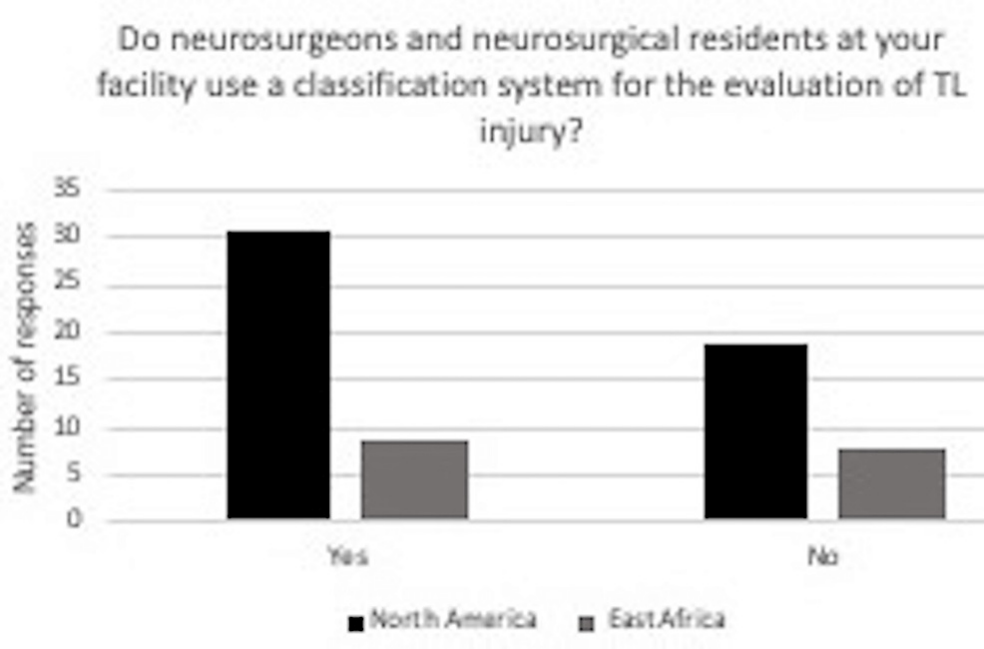
In response to the question “Do neurosurgeons and neurosurgical residents at your facility use a classification system for the evaluation of thoracolumbar injury?” for surgeons in North America, 31 said yes, and 19 said no. In East Africa, nine surgeons said yes, and eight surgeons said no.

Results became more pronounced when evaluating reliance on the classification systems to guide the management of thoracolumbar spine injury, shown in Figure 4. In North America, 14 out of 50 (28%) reported greater than 80%. For respondents practicing in East Africa, 75% reported greater than 80%, 12.5% reported between 60-80%, and 12.5% reported between 40-60%. Many surgeons in North America reported that their medical trainees at their facility used a classification system for the evaluation of the thoracolumbar injury, as 62% responded yes to the survey question. In East Africa, 56% of surgeons responded that their medical trainees used a classification system to evaluate thoracolumbar injury (Figure 5).

**Figure 6:**
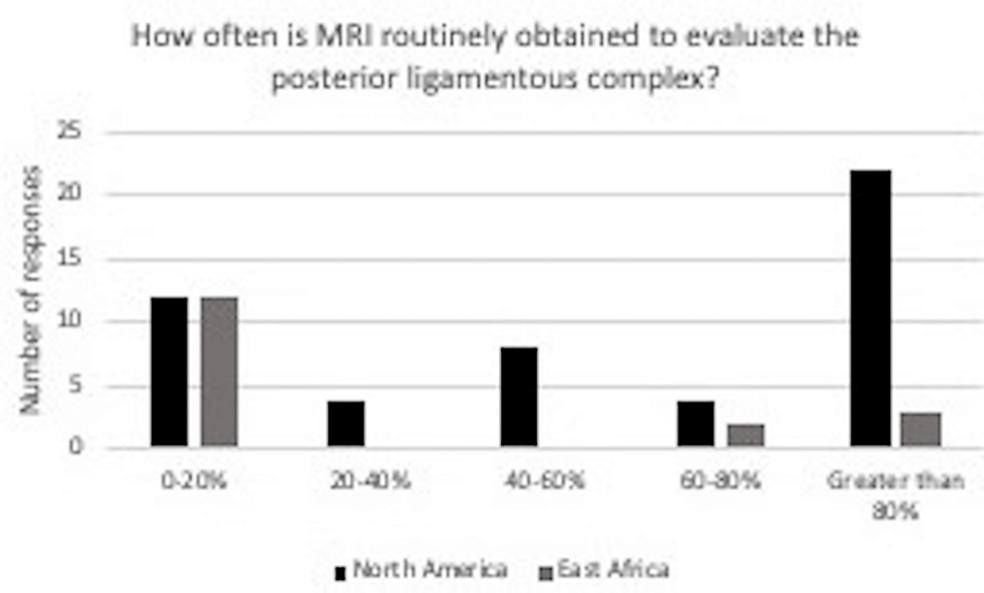
In response to the question “How often is MRI routinely obtained to evaluate the posterior ligamentous complex?” for those in North America, 12 said 0-20%, four said 20-40%, eight said 40-60%, four said 60-80%, 22 said greater than 80%. For those in East Africa, 12 said 0-20%, 0 said 20-40%, 0 said 40-60%, two said 60-80%, three said greater than 80%.

Figure 6 demonstrates notable differences in MRI routine use in evaluating the posterior ligamentous complex in spine trauma patients. In North America, 44% of surgeons reported regular use of MRI in greater than 80% of spine trauma cases. For East Africa, 68.7% of surgeons reported regular use of MRI in evaluating the posterior ligamentous complex in 0-20% of spine trauma cases.

**Figure 7:**
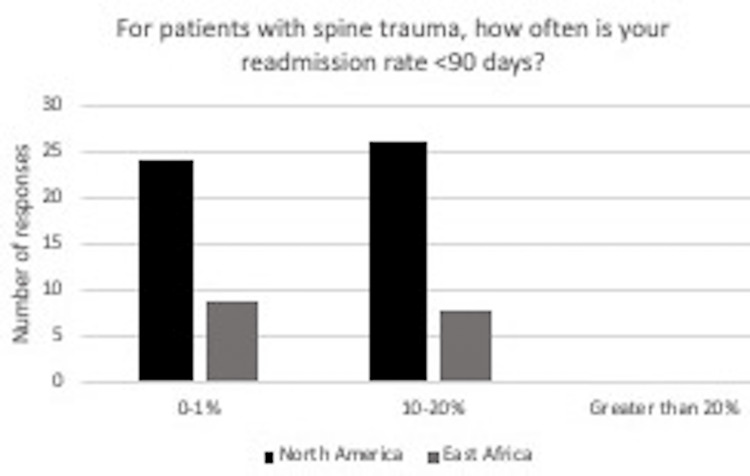
In response to the question, “For patients with spine trauma, how often is your readmission rate <90 days?” for those in North America, 24 said 0-1%, 26 said 10-20%, and 0 said greater than 20%. For those in East Africa, nine said 0-1%, eight said 10-20% and 0 said greater than 20%.

Interestingly, the reported readmission rate of <90 days for patients with spine trauma was similar among surgeons in North America and Africa. For surgeons in North America, 52% reported a readmission rate of <90 days in 10-20% of cases. In East Africa, surgeons reported a 47% readmission rate of <90 days in 10-20% of cases (Figure 7).

## Discussion

Our survey results showed more significant similarities in evaluating thoracic and lumbar spine injury secondary to trauma in patients among regions than we expected. The remarkable difference between surgeons in East Africa and North America was the difference in the type of classification system used and the routine use of MRI in the evaluation of thoracolumbar spine trauma. Notable was surgeons in East Africa, and North America reported a similar percentage of readmission <90 days.

In Figure 2, nine (or 53%) East African respondents reported approximately 20-40% of thoracolumbar spine fractures at their facility. In contrast, 15 (or 30%) North American surgeons reported about 20-40% of thoracolumbar spine fractures at their facility. A total of 21 (or 42%) North American surgeons reported an approximate percentage of thoracolumbar spine fractures at their facility ranging from 0-20%. A total of 0 surgeons in East Africa reported an approximate rate of thoracolumbar spine fractures in this percentage range of 0-20%. A systematic review of 64 studies from 28 developing countries found the two leading causes of spinal cord injury (SCI) were motor vehicle crashes (41.4%; 95% CI: 35.4-47.4) and falls (34.9%; 95% CI: 26.7-43.1) [8]. Although spine fractures represent only a minority in all trauma patients, their influence on the patient's social and financial environment is more significant than other injuries [9].

Notable are the limitations and disparities between the developed and developing world's ability to provide acute care are evident immediately following an SCI. In low-resource countries, individuals with acute SCI are unlikely to be fully immobilized in the field and transported by trained healthcare professionals [10]. In cases of spinal instability, further neurological comprise often occurs. For specialized care readily available, delays are common between the initial injury and the presentation time for specialized care. A study completed in India found an average 45-day delay between injury and presentation to the spinal care unit, primarily due to a lack of knowledge by healthcare providers that spinal care units existed [11].

A notable difference was using different classification systems among surgeons in North America and East Africa, as shown in Figure 3. Eleven (or 65%) surgeons practicing in East Africa reported using the TLICS scoring system in evaluating thoracolumbar spine injury, and 31 (or 62%) surgeons practicing in North America reported using the Denis 3-column classification system. The Thoracolumbar Injury Classification and Severity (TLICS) Score falls short in clearly defining management for patients with stable burst fractures who are neurologically intact, including patients with a "grey zone" score of four points. The patients in this intermediate group present with varied fracture patterns and neurologic status with recommended treatment options of either operative or conservative interventions.

As there is no acceptance of a universal TL spinal injury classification system, it is imperative to understand the evolution of spinal injury classification. At the University of Missouri in Columbia, Missouri, where both authors currently reside, most thoracolumbar spine injuries are evaluated using the AO Spine Thoracolumbar Injury Classification System. Figure 5 highlights the use of classification to evaluate thoracolumbar injury by medical trainees. In North America, 31 surgeons (or 62%) reported that medical trainees at their facility used a classification system to evaluate thoracolumbar spine injury, and nine (or 52%) surgeons in East Africa.

Establishing a classification system used to evaluate spinal trauma provides effective communication between treating personnel, guide management, and estimate the patient's prognosis. Unfortunately, most classification systems cannot provide a simple, comprehensive, reproducible, and reliable scoring metric. The currently used classification systems are too complex for routine or acute care settings and too inclusive.

While there is no current universal acceptance of a classification system for thoracolumbar injury, East African surgeons have a significant reliance on a classification system in managing spine injuries, as demonstrated in Figure 4. A total of 13 (or 76%) surgeons in Africa reported that greater than 80% of their cases rely on a classification system to guide the management of patients with thoracolumbar injury. In North America, 14 (or 28%) surgeons reported greater than 80% of the cases, and a classification system is used to manage thoracolumbar injury.

A remarkable finding in our study was the variability in responses from surgeons in East Africa and North America regarding the routine use of MRI in evaluating the posterior ligamentous complex in spine trauma patients, as shown in Figure 6. Most surgeons in East Africa reported less common use of MRI in evaluating the posterior ligamentous complex, as 12 (or 71%) surgeons in East Africa reported the use of MRI in 0-20% of spine trauma cases. In contrast, 22 (or 44%) surgeons in North America reported the use of MRI in >80% of spine trauma cases, and three (or 18%) surgeons reported the use of MRI in >80% of spine trauma cases. These differences highlight the variability in resources available to surgeons on different continents, in this case, North America and East Africa. In spinal cord edema, contusion, hemorrhage, and ischemia, MRI findings may serve as prognostic indicators [12].

Imaging is critical in evaluating acute spinal trauma and helps provide prompt and accurate treatment to patients. Due to its increased availability in the emergency setting and superior contrast resolution, the spine's stability may be assessed using CT for surgical decision-making by neurosurgeons. Although, the literature reports that the use of MRI has played a crucial role in evaluating and managing spine trauma patients [13]. MRI is the modality of choice for assessing soft tissue, spinal cord, ligamentous and osseous injuries [14]. A TLICS gray zone shortcoming was related to the disagreement between treating surgeons on the integrity of the PLC. Definite criteria for PLC injury may be necessary, while differentiating PLC injury between TLICS scores 0, 2, and 3 is complicated [15].

Another remarkable finding in this study was the reported similar readmission rate of <90 days among neurosurgeons in East Africa and North America. As Figure 7 demonstrates, 26 (or 52%) respondents in North America reported a readmission rate ranging from 10 to 20%, whereas eight (or 47%) respondents in North America reported a readmission rate ranging from 10 to 20%. Surgeons in East Africa and North America reported 0 readmissions <90 days in greater than 20% of cases. The responses to our survey demonstrated that readmission rates were low, which is encouraging and demonstrate more significant advances in spine trauma classification for East African patients than expected.

Previous studies have reported in resource-rich countries that most individuals survive the 12 months following their injury; however, there is a marked discrepancy in observed mortality between resource-rich and resource-poor environments. A study of 24 individuals from Sierra Leone reported that seven individuals died during the initial hospitalization, had an additional eight mortalities at follow-up (average 17.4 months), and lost four to follow-up [16]. Two out of the five survivors were ambulatory due to incomplete injuries. Furthermore, an additional review reported a two-fold difference between the highest-reported mortality rate in a developing country (17.5 annual deaths attributable to SCI per million people in Nigeria) vs. that in a developed country (eight yearly deaths attributable to SCI per million people in Canada) [2].

The study presented showed a low response rate among neurosurgeons, primary neurosurgeons residing in North America. Forty-one emails were sent to surgeons in East Africa and 399 in North America. The response rates were 41.5% and 12.5%, respectively. East African surgeons had a response rate of 3.32 times greater than North America. The low response rate is likely due to various factors, including outdated email addresses listed on the AANS directory, demanding workload, or decreased interest in completing academic surveys. Surgical workload may be a significant factor. A study by Abebe et al. from 2007-2008 reviewed the pattern of neurosurgical procedures in Ethiopia. It revealed the limited operative capacity of our neurosurgical service, as demonstrated by the number of neurosurgical procedures over the two years, which was only 1364.18. When calculated, this means surgeons performed only one operation per day at either of the two teaching hospitals [17]. The findings in this study prompted advancements and changes in neurosurgical care in Ethiopia; thus, it is difficult to determine if the workload is the primary determinant for the survey responses in this study. For future academic research, it would be beneficial to evaluate further the reasons for low survey response rates among surgeons in North America. The low response rate in this study may account for the possibility of bias.

The strength of this study was that many neurosurgeons were invited to participate in this survey, including multiple geographic areas. The survey included surgeons from the United States and East Africa. By distributing the survey on a continental level, the authors could better understand the standard of care in different clinical settings, which may be markedly different in certain regions. For future work, our study questionnaire could be tailored to specific regional practice outcomes to evaluate neurosurgeons' differences more closely. This would allow the authors to direct the questions toward surgeons in a particular country, state, or city to evaluate thoracolumbar spine injury classification.

The limitations of our study include decreased response rates from neurosurgeons, which may bias the results. Additionally, we could not identify standards of care in specific countries included in the survey. Compared to Guinea or Zanzibar, outcomes for patients undergoing surgery in Ethiopia may differ substantially due to patient load, education, and resources. A clearer understanding of these differences and why such differences exist is a probable next step for the authors. It would help develop methods more adequately to alleviate spine trauma care limitations and improve patient outcomes.

## Conclusions

This study's reported findings from surgeons practicing in North America and East Africa highlight the variability in the classification system used to evaluate thoracolumbar spine injuries. Improving access to neurosurgical training programs in East Africa may further educate training neurosurgeons in evaluating thoracolumbar spine injury. In our study, there was a higher percentage of reported thoracolumbar spine fractures in East Africa. However, fewer neurosurgeons from East Africa reported using a classification system to evaluate spine trauma patients compared to neurosurgeons in North America. An interesting finding was the reported readmission rate of <90 days among neurosurgeons in North America and East Africa. However, further evaluation of the cause of readmission rate should be evaluated to determine the cause of readmission <90 days. The findings of this study highlight the variability of thoracolumbar classification system type in the evaluation of thoracolumbar spine trauma patients among neurosurgeons and neurosurgical residents in North America and East Africa.
